# A Scoping Review: Is Yoga an Effective Intervention for Erectile Dysfunction and Premature Ejaculation?

**DOI:** 10.7759/cureus.53265

**Published:** 2024-01-30

**Authors:** Subrahmanaya Bhat, Manoj K Pandey, Udayakumar K, Nikunj Gokani, T.S. Sathyanarayana Rao

**Affiliations:** 1 Yoga, JSS Academy of Higher Education and Research, Mysore, IND; 2 Clinical Psychology, JSS Medical College, JSS Academy of Higher Education and Research, Mysore, IND; 3 Human Consciousness and Yogic Sciences, Mangalore University, Mangalagangothri, IND; 4 Psychiatry, JSS Medical College, JSS Academy of Higher Education and Research, Mysore, IND

**Keywords:** anxiety, depression, premature ejaculation, erectile dysfunction, yoga

## Abstract

There is increasing concern among both healthcare professionals and the general public about the long-term effectiveness and possible adverse effects of medicines used to treat premature ejaculation (PE) and erectile dysfunction (ED). There is also a growing recognition of the advantages of incorporating alternative or traditional approaches into healthcare systems. Yoga is gaining popularity globally and has emerged as a viable adjunct and alternative to add value to patient care and prevention of illnesses, which requires further investigation. This scoping review aimed to explore the effects of yoga as an independent or adjunct intervention in treating ED and PE.

In this review study, researchers conducted a systematic literature review from 2000 to 2023 as per the Preferred Reporting Items for Systematic Reviews and Meta-Analyses (PRISMA) guidelines. Electronic databases of Scopus, Google Scholar, Web of Science, and PubMed were used for literature searches. Studies published in the English language on male individuals with ED and PE and those with comorbid stress, anxiety, and depression were also included. Studies on these sexual dysfunctions, comorbid with HIV/AIDS, and severe psychiatric conditions, i.e., schizophrenia, bipolar affective disorders, and substance dependence, except alcohol, were excluded. Ten studies out of the 2016 selected articles met the inclusion criteria and were included in the final analysis.

The findings of this scoping review revealed that yoga interventions are more effective in managing PE and ED, with a greater emphasis on the former. Yoga is an effective, safe, and affordable approach recommended for managing erectile functions and PE. Men can improve their quality of life and regain confidence in sexual functioning by incorporating yoga into their routines. The study shows the potential benefits of yoga for both conditions, indicating the need for further robust studies in this area. Researchers advocate practising yoga under professional supervision for optimal safety and guidance.

## Introduction and background

The Diagnostic and Statistical Manual of Mental Disorders, 5th Edition, defines erectile dysfunction (ED) as the recurrent inability to achieve, maintain, or decrease erectile rigidity during partnered sexual activity [1]. ED is often linked to psychological or physiological disorders (e.g., alcohol dependence, hypertension, and diabetes), resulting in stress, depression, and anxiety. Performance anxiety arises from difficulty maintaining an erection. A five-item International Index of Erectile Function (IIEF-5) questionnaire is widely favoured to evaluate ED [2,3]. ED is primarily classified into two types: organic and non-organic. A combination of endocrine and non-endocrine factors can cause organic ED, i.e., hormonal abnormalities impact libido, while vascular insufficiency and nerve injury in the penis can disrupt blood flow. Men are more likely to have ED if they have medical conditions like diabetes or experience adverse effects from certain drugs, particularly antidepressants [4-6].

ED is a condition that commonly affects people worldwide and becomes more prevalent with age. It is estimated that around 1-10% of people under 40 years have ED, while 20-40% of those aged 60-69 years are affected [7]. In Asia, the prevalence ranges from 9% to 73% [8]; however, these rates could be influenced by varying assessment methods and types of surveys. Pharmacological treatments of ED, like sildenafil and phosphodiesterase 5 (PDE5) inhibitors, can have adverse effects, and there are concerns about the sustainability of treatment. Identifying risk factors is recommended for appropriate treatment [9,10].

Premature ejaculation (PE) is characterized by recurrent ejaculation within one minute after vaginal penetration, causing distress to the individual, and is prevalent in 31% of men aged 18-59 years globally [1,11]. Psychological factors and medical conditions contribute to primary and secondary PE [12]. PE is usually evaluated through intravaginal ejaculation latency time (IELT), measured from penetration to ejaculation [13]. Among other associated factors, primary PE can often be linked to psychological conditions like anxiety disorders, depression, stress, and partners' interpersonal conflicts. Secondary PE can be usually triggered by diabetes, hypertension, hyperthyroidism, alcoholism, or substance abuse [12].

It is not uncommon for both ED and PE to occur together. Although medications are available to manage these conditions, as mentioned above, they can have potential side effects and have concerns about the durability of treatment outcomes. Studies have shown that lifestyle modifications can play a crucial role in managing and preventing both ED and PE [14], and non-pharmacological treatments and preventive methods, such as yoga, may offer a promising alternative. The effectiveness of yoga in managing and preventing ED and PE has been subject to mixed findings in existing literature. Therefore, a scoping review of the literature was planned to explore the effectiveness of yoga on these conditions, incorporating the recent recommended guidelines for review studies. Thus, this review aims to investigate the potential of yoga as a non-pharmacological intervention for ED and PE.

## Review

Methods

Search Strategy

An electronic literature search was conducted in Scopus, Google Scholar, Web of Science, and PubMed databases. The studies sought were from January 2000 to February 2023. The search was carried out using a combination of keywords such as [Yoga[tab] OR Yoga OR yogic OR yog∗ OR “yogasana” OR “Surya namaskar” OR “Thai yoga” OR “asana” OR “hatha” OR “pranayama” OR “pranayam” OR “dhyana” OR “meditation” OR “lifestyle modifications”] AND [“erectile dysfunction” OR “sexual dysfunction” OR “sexual disorder” OR “premature ejaculation” OR “sexuality” OR “infertility”] AND [“stress” OR “depression” OR “anxiety”].

Selection Process

Inclusion criteria: This review included studies published in English, including original and review articles on male patients with ED and PE. Patients with these disorders who had comorbid conditions such as stress, anxiety, or depression were also included, as these conditions are quite prevalent in patients with ED and PE.

Exclusion criteria: The study did not consider research articles unavailable in full text. Moreover, it excluded studies related to patients with comorbid conditions such as HIV/AIDS and severe psychiatric conditions like schizophrenia, bipolar affective disorders, and substance dependence, except for alcohol dependence, which is closely associated with ED and PE.

Data Collection Process

**Figure 1:**
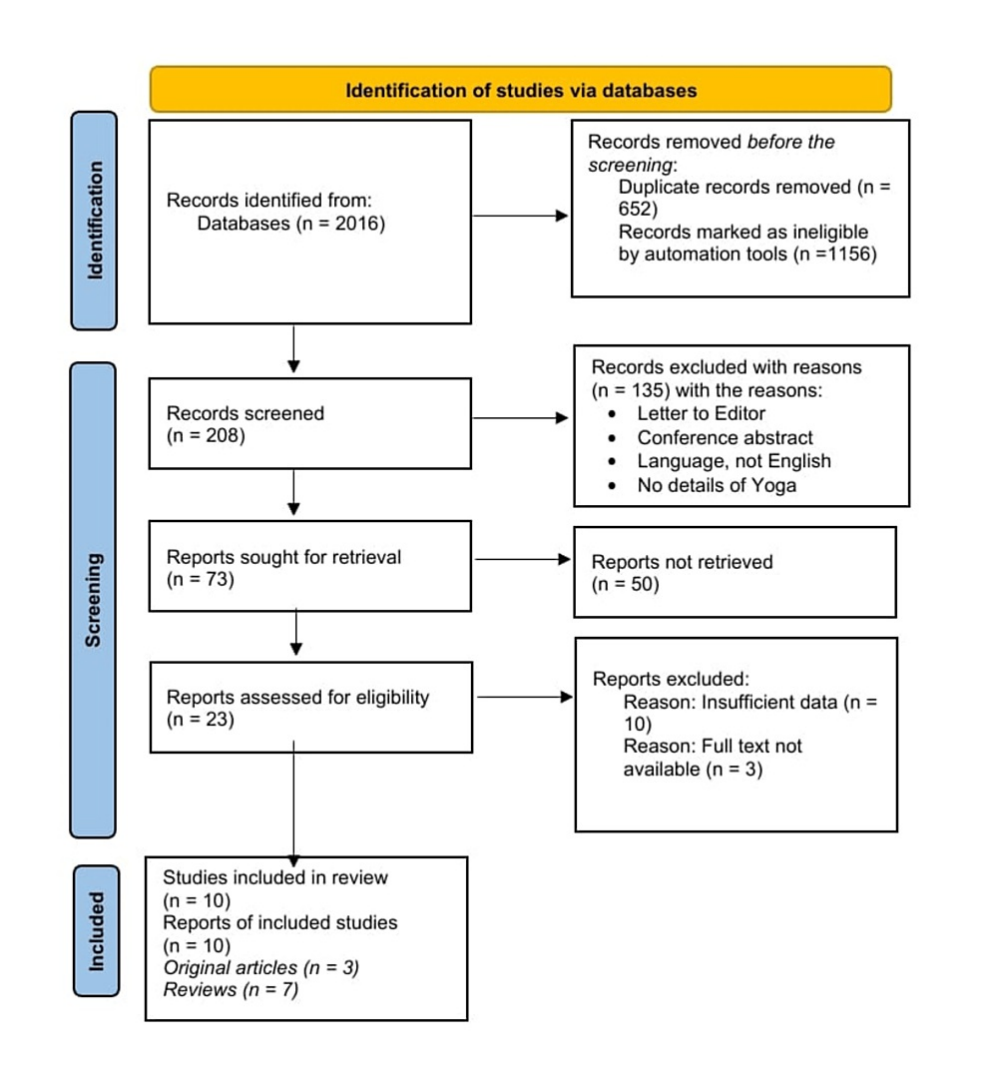
PRISMA flow diagram of the review study selection process PRISMA: Preferred Reporting Items for Systematic Reviews and Meta-Analyses.

The Preferred Reporting Items for Systematic Reviews and Meta-Analyses (PRISMA) guidelines [15] were applied to carry out this study. After conducting a literature search of 2016 articles by one author, a team of two authors screened and selected 10 papers based on inclusion criteria. Discrepancies were resolved through discussion with a fourth author. PRISMA flow diagram followed for identification, screening, and inclusion of data is given in Figure 1.

Results

In the current review, the analysis of the experimental studies demonstrates that two experiments [16,17] compared the use of yoga poses and medications for PE through the IELT (Table 1). The yoga asanas selected were targeted at strengthening the muscle and plasticity of the pelvic region and perineal muscles to improve blood circulation and aid contraction. Both studies observed that the yoga groups showed significant improvement in IELT and overall sexual experience compared to the control group. Outcomes of a non-comparative pilot study (Table 1) favoured yoga intervention where all 65 males experienced a significant improvement in ejaculation control and erectile quality [18].

**Table 1:** Details of original articles examining the effect of yoga on male sexual functioning PE: premature ejaculation.

Author, year, & title	Studied population, groups, study design	Intervention & techniques	Measure time points	Main findings
Author: Dhikav et al. (2007) [16]. Title: Yoga in premature ejaculation: a comparative trial with fluoxetine	Out of 68 patients with PE, 38 were in the yoga group with a mean age of 38.9 (10.10) years and 30 were in the control group (fluoxetine group) with a mean age of 38.6 (9.20) years. Design: comparative trial	The yoga group practised 12 asanas and two pranayamas for an hour daily. The control group was allocated with fluoxetine capsules in a single dose of 20-60 mg daily. Intravaginal ejaculatory latencies were taken and evaluated after eight weeks.	12 weeks	All 38 patients in the yoga group showed statistically significant improvement in PE, with 65.7% reporting good and 34.2% reporting fair improvement. In the control group, 25 out of 30 patients (83%) experienced significant improvement in PE.
Author: Rohilla et al. (2020) [17]. Title: A comparative study of yoga with paroxetine for the treatment of premature ejaculation: a pilot study	68 male patients with problems of early ejaculation. 40 patients were selected who were on paroxetine, mean age = 30.38 (4.79), and 28 chose yoga treatment, mean age = 31.36 (4.77). Design: nonrandomized, nonblinded comparative study in a tertiary care centre	First two weeks, both groups were taught the process of using intravaginal ejaculation latency time (IELT). The yoga group was provided photos of yoga postures as well as audio-visual instructions on mobile by the trainer. Patients underwent at least three measurements at four-week intervals throughout the 12-week intervention. During these assessments, the IELT was documented, and any treatment-related side effects were recorded.	6, 10 and 14 weeks	62 patients completed the study. Six patients were excluded due to non-compliance. IELT significantly increased in both groups: paroxetine (from 29.85 ± 11.9 to 82.19 ± 32.9) and yoga (from 25.88 ± 16.1 to 88.69 ± 26.9). Yoga had a slightly delayed onset effect, but its effect size (η2 = 0.87, p < 0.05) was significantly larger than paroxetine's (η2 = 0.73, p < 0.05). 19.5% of the paroxetine group and 8% of the yoga group continued to have PE issues at the end of the trial. The yoga treatment group reported better improvement than the paroxetine group.
Author: Dhikav et al. (2010) [18]. Title: Yoga in male sexual functioning: A noncomparative pilot study	65 males diagnosed with premature ejaculation, aged between 24 to 60 years. Design: non-comparative pilot study	A questionnaire based on Male Sexual Quotient (MSQ) before and after yoga camp. Participants were told to perform yoga asanas that have beneficial effects on abdominal-pelvic muscle tone, gonads, endocrines, digestion, and joint movements with a yoga instructor for one hour each day. Breathing techniques were also enforced.	12 weeks	After doing yoga for 12 weeks, the study found a significant overall improvement of 11.81% (p < 0.0001) in sexual function scores as determined by the MSQ. Every category showed improvement, although intercourse satisfaction (24.6%), ejaculation control (20.6%), and erection quality (14.15%) showed the most gains. Maintenance of erection during intercourse (16.9%), on-demand erection (13.2%), and hardness (12.6%) were the subdomains of erection quality that improved in the order mentioned. Following the yoga intervention, there were notable improvements in the physical and emotional components of sexual experiences, such as desire, confidence, intercourse satisfaction, erection, control over ejaculation, and orgasm.

Further, seven review studies (Table 2) provide evidence that yoga may be an alternative therapy for men with PE and other sexual dysfunctions. Four other studies [19-22] mainly discussed the mechanism and benefits of some yoga poses, pranayama, and breathing techniques that improve PE, sex life, and performance. Finally, three articles were reviewed and highlighted the effect of yoga intervention on infertility [23-25]. Results of these review studies showed that yoga improves sperm count, motility, and prostate secretion in men. It potentially boosts fertility by stimulating hormone levels, improving blood and nutrient supply, and regaining sexual stamina, which can help men facing ED and PE.

**Table 2:** Details of review articles examining the effect of yoga on male sexual functioning ED: erectile dysfunction; ART: assisted reproductive technology.

Author, year, & title	Background	Method	Main findings
Author: Rakshith et al. (2017) [19]. Title: Yogic intervention in sexual dysfunction - a review	This review explores the mechanisms and benefits of standing, seated, supine, and inversion yoga poses, alongside Mudra and Bandha practices, emphasizing their diverse therapeutic advantages for sexual dysfunction.	Reviewed literature on original articles and review articles by utilizing a qualitative review style.	Seated supine and inversion yoga poses stimulate hormones, improve blood circulation, and strengthen reproductive organs, while also reducing stress, anxiety, and fatigue. Mudras can help balance testosterone levels, encourage sperm count, and control premature ejaculation. Moola Bandha helps increase awareness of genital arousal sensations.
Author: Joshi et al. (2020) [20]. Title: Role of yoga in the management of premature ejaculation, male sexual health and dysfunction	This review explores the role of yoga in managing premature ejaculation, analysing the mechanics of various yoga components, including asanas, cleansing techniques, breathing exercises, mudra, and bandha.	Reviewed literature on original articles and review articles by using a qualitative review style.	Seated poses improve pelvic health, tone reproductive organs, and enhance the urinary system, leading to better arousal and orgasm control. Pranayama reduces blood pressure and enhances cerebral blood flow and oxygenation.
Author: Sengupta et al. (2013) [21]. Title: Male reproductive health and yoga	This review examines how yoga practices positively impact reproductive health, focusing on mechanisms that may prevent infertility. It provides essential insights into the beneficial effects of yoga on fertility.	Reviewed non-empirical and empirical pieces of literature.	Regular yoga practice can reduce stress and anxiety levels, improve sex life, and help treat mild ED issues. Yoga asanas are recommended at various life stages to enhance strength, blood flow, and sexual desire and alleviate stress, depression, and performance anxiety. Yoga can be beneficial in the prevention of infertility and improve male reproductive health.
Author: Brotto et al. (2009) [22]. Title: Yoga and sexual functioning: a review	This review examines the literature on yoga philosophy and its various forms, evaluating nonempirical and limited empirical links between yoga and improved sexual function. It proposes future research paths, emphasizing yoga as a potential treatment for sexual complaints.	Reviewed non-empirical and empirical pieces of literature.	Yoga strengthens and tones the urogenital area and strengthens pelvic floor muscles, which results in increased blood flow in the genitals. Moola Bandha shows promising results in the treatment of sexual disorders. Pranayama and meditation enable relaxation and lower stress and performance anxiety. Kundalini yoga has great potential in the treatment of sexual problems with its focus on sexual energy.
Author: Kochhar et al. (2017) [23]. Title: The role of traditional diet and yoga for infertility: a blend and balance of traditional knowledge and modern medicine	This review explores yoga's potential in promoting reproductive health and treating infertility. By analysing the benefits of yoga practices, it highlights their efficacy in improving sexual function and overall well-being.	Reviewed literature on on-empirical theories.	Yoga can help regulate the hypothalamic-pituitary-gonadal axis, balance hormone levels, reduce stress and anxiety, and improve overall quality of life. Additionally, it helps in treating mild ED. Incorporating yoga into a fitness routine can positively impact fertility by enhancing hormone levels and blood circulation to the reproductive system. This can help improve sexual stamina and increase chances of conceiving. The study provides insights into yoga's use as a complementary therapy for reproductive health issues by examining relevant literature.
Author: Darbandi et al. (2018) [24]. Title: Yoga can improve assisted reproduction technology outcomes in couples with infertility	The study investigated the effects of yoga, including asanas (yoga poses), pranayama (proper breathing), Shavasana, and meditation, on male and female fertility as well as ART outcomes.	Reviewed literature on non-randomized or uncontrolled trials.	Yoga practices can improve sperm count, motility, and prostate secretion in men. Additionally, yoga has been found to enhance immune system disorders, varicocele, and intravaginal ejaculation time while reducing sexual dysfunction. Furthermore, Moola Bandha, a specific yoga practice, has been shown to help with spermatorrhea, prevent inguinal hernia, and regulate testosterone secretion. It may also increase genital arousal sensations and potentially help with premature ejaculation.
Author: Shobitha et al. (2016) [25]. Title: Impact of yoga on mind-body management and its possible scientific mechanisms	This article examines the scientific basis of yoga and its positive impact on health. It provides a holistic view of the health benefits by discussing how yoga influences various body systems.	Reviewed original articles and review articles by utilizing a qualitative review style.	Regular yoga practices improve sperm count and motility in men. It also improves overall sexual function and helps to treat mild ED. Yoga improves reproductive health by reducing stress and anxiety and improving autonomic functions by suppressing sympathetic activity and promoting parasympathetic activity.

Discussion

Yoga is a holistic approach that potentially complements medical treatments for ED and PE. It involves physical postures, breathing exercises, mindfulness, and meditation that promote overall health and positively impact sexual health and function. Yoga’s integrative nature makes it an alternative option for managing ED and PE. Studies compared the experiential effects of yoga asanas to drug treatments for PE and revealed that the yoga intervention groups significantly improved IELT and erection quality [16,17]. In these studies, practising yoga postures strengthened the muscles in the pelvic and perineal regions, improved blood circulation, and enhanced contraction. These targeted mechanisms are believed to contribute to the observed improvements in ED and PE functions. These findings suggest that yoga has the potential to be an effective and accessible alternative for managing PE and ED. Another non-comparative pilot study [18] has shown that yoga has a substantial effect on enhancing sexual functioning. The study involved 65 males who participated in dedicated yoga sessions for 12 weeks. After the sessions, significant improvement was observed in their Male Sexual Quotient (MSQ) scores, i.e., improvement in intercourse satisfaction, ejaculation control, and erection quality. This not only validates the practical benefits of yoga in addressing sexual dysfunctions but also highlights the overall impact of yoga on various aspects of sexual well-being.

Yoga has numerous benefits for reproductive health. It can help stimulate hormones, improve blood circulation, and strengthen reproductive organs [23], which in turn helps address sexual function problems. Specific practices such as mudras and Moola Bandha can help achieve hormone balance and pelvic awareness, both of which show promise in treating sexual disorders. Seated poses and pranayama can improve pelvic health, reduce stress, and enhance arousal control [21-24]. Regular yoga practice can positively impact fertility by regulating hormonal balance, reducing stress, improving blood circulation and sexual stamina, and increasing chances of conception, thus improving overall quality of life.

Additionally, yoga was found to increase sperm count, motility, prostate secretion, and overall sexual function, making it a valuable tool for reproductive health [20,24,25]. The study explored the benefits of yoga intervention in adults with infertility and assessed the effects through fertility rates and outcomes of assisted reproductive technology (ART). The results of the study show that practising yoga can potentially enhance fertility by improving sperm count, motility, and prostate secretion in men [24].

ED can cause low confidence and low self-esteem, which are linked to depression, stress, and anxiety. Yoga can improve muscular strength, flexibility, and respiratory and cardiovascular function, help reduce the severity of stress, anxiety, and depression, and improve mental well-being [26-28]. Adding to the ED and PE studies, researchers are now exploring the relationship between male infertility and sexual dysfunction. Studies conducted by Liu et al. (2022) have revealed a strong correlation between male infertility and sexual dysfunctions such as ED and PE. Other non-controlled studies have also reported sexual dysfunction in infertile men [29,30]. This highlights the potential of yoga intervention as a viable adjunct and alternative not only for ED and PE but also for men with infertility issues. Thus, in a comprehensive management of ED and PE, adjunct to pharmacological treatment, the yoga intervention may substantially improve illness and enhance the patient's quality of life.

Limitations

This scoping review selected literature published in English, and including other languages may provide additional data and potentially provide more decadent literature in the area. A literature search in the electronic databases yielded a few empirical research articles, most of which were reviews. Moreover, there are limited studies on the effects of yoga, specifically on ED. The current review attempted to provide a glimpse into the impact of yoga on ED and PE, which further calls for more comprehensive quantitative reviews and experimental studies to reach a conclusive finding about the effects of yoga on these two most prevalent male sexual dysfunctions.

## Conclusions

In summary, despite its inherent limitations, the present scoping review demonstrates that yoga is a viable, safe, and cost-effective comprehensive treatment plan for managing ED and PE. Yoga is a holistic practice that includes physical postures, breathing exercises, mindfulness, and meditation. Each component has multidimensional effects on various bodily systems, including organ-specific benefits. The regular practice of yoga can bring about several benefits for male sexual health. It promotes blood circulation, strengthens the pelvic and perineal regions, boosts penile muscle contraction, improves arousal control, and enhances sexual stamina, prostate secretion, and pelvic awareness. These mechanisms contribute to the improvement of ED and PE. Yoga, apart from its physiological benefits, has been observed to have a positive impact on mental health by reducing symptoms of stress, anxiety, and depression. These conditions are reported to affect sexual health indirectly, and yoga addresses them, which also adds to the improvements in PE and ED.

In essence, yoga has emerged as an integrated, comprehensive, adjunct, and alternative approach to health and well-being. There have been worldwide efforts to integrate yoga into the management of mental health issues, including male sexual dysfunctions. Moreover, this scoping review underscores the need for multicentric random control trials and longitudinal cohort studies to limit methodological discrepancies and generate a more robust database providing credible, validating findings on yoga's effectiveness in treating male sexual dysfunctions.
